# Microbiology Clinical Culture Diagnostic Yields and Antimicrobial Resistance Proportions before and during the COVID-19 Pandemic in an Indian Community Hospital and Two US Community Hospitals

**DOI:** 10.3390/antibiotics12030537

**Published:** 2023-03-08

**Authors:** Sumanth Gandra, Gerardo Alvarez-Uria, Dustin Stwalley, Katelin B. Nickel, Kimberly A. Reske, Jennie H. Kwon, Erik R. Dubberke, Margaret A. Olsen, Jason P. Burnham

**Affiliations:** 1 Department of Internal Medicine, Division of Infectious Diseases, Washington University in St. Louis School of Medicine, 4523 Clayton Avenue, Campus Box 8051, St. Louis, MO 63110, USA; 2 Department of Infectious Diseases, Rural Development Trust Hospital, Bathalapalli, Anantapur 515661, India

**Keywords:** COVID-19, microbiology, antimicrobial resistance

## Abstract

Studies comparing the impact of the COVID-19 pandemic on diagnostic microbiology culture yields and antimicrobial resistance proportions in low-to-middle-income and high-income countries are lacking. A retrospective study using blood, respiratory, and urine microbiology data from a community hospital in India and two community hospitals (Hospitals A and B) in St. Louis, MO, USA was performed. We compared the proportion of cultures positive for selected multi-drug-resistant organisms (MDROs) listed on the WHO’s priority pathogen list both before the COVID-19 pandemic (January 2017–December 2019) and early in the COVID-19 pandemic (April 2020–October 2020). The proportion of blood cultures contaminated with coagulase-negative *Staphylococcus* (CONS) was significantly higher during the pandemic in all three hospitals. In the Indian hospital, the proportion of carbapenem-resistant (CR) *Klebsiella pneumoniae* in respiratory cultures was significantly higher during the pandemic period, as was the proportion of CR *Escherichia coli* in urine cultures. In the US hospitals, the proportion of methicillin-resistant *Staphylococcus aureus* in blood cultures was significantly higher during the pandemic period in Hospital A, while no significant increase in the proportion of Gram-negative MDROs was observed. Continuity of antimicrobial stewardship activities and better infection prevention measures are critical to optimize outcomes and minimize the burden of antimicrobial resistance among COVID-19 patients.

## 1. Introduction

Antimicrobial resistance is a global public health problem, with higher prevalence and burden in low-to-middle-income countries (LMICs) when compared with high-income countries (HICs), even prior to the coronavirus disease 2019 (COVID-19) pandemic [1]. In 2017, the World Health Organization (WHO) published an antibiotic-resistant priority pathogen list that included those organisms posing the greatest threats to human health [2]. Multi-drug-resistant organisms (MDROs), e.g., carbapenem-resistant *Acinetobacter baumannii*, carbapenem-resistant *Pseudomonas aeruginosa*, third-generation cephalosporin- and carbapenem-resistant *Enterobacterales*, methicillin-resistant *Staphylococcus aures*, and vancomycin resistant *Enterococcus faecium*, which are typically associated with healthcare-acquired infections (HAIs), are included on this list. Patients hospitalized with severe acute respiratory syndrome coronavirus 2 (SARS-CoV-2) are at increased risk of contracting HAIs, with MDROs resulting from prolonged hospital stays due to the presence of mechanical devices, exposure to broad-spectrum antibiotics, and sub-optimal infection control practices [2,3,4,5,6,7,8,9]. However, variations in microbiological diagnostic test utilization practices, empiric antibiotic choices, and infection control practices between LMICs and HICs could have a differential impact on antimicrobial resistance rates among COVID-19 patients. Limited studies have examined the impact of the COVID-19 pandemic on diagnostic microbiology cultures [10,11,12,13,14,15,16,17,18,19,20], but all except two were performed in HICs [10,19], and the majority of the studies only examined blood cultures [11,12,13,14,15,16,19,20]. Studies examining the impact of the COVID-19 pandemic on diagnostic microbiology cultures simultaneously in a LMIC and HIC are needed to better understand the impact of socioeconomic and cultural differences on healthcare outcomes.

In this study, we sought to evaluate the diagnostic yields of blood, respiratory, and urine cultures as well as the isolation of MDROs listed in the WHO’s priority pathogen list both before and during the COVID-19 pandemic in a community hospital in India and in two community hospitals in the United States.

## 2. Results

A total of 7660 specimens (blood, respiratory, and urine) were obtained from inpatients at the Indian hospital during the study period (pre-pandemic period: 6763; pandemic period: 897), 40,163 specimens were obtained from Hospital A (pre-pandemic period: 38,555; pandemic period: 1608), and 43,831 specimens were obtained from Hospital B (pre-pandemic period: 43,238; pandemic period: 548), respectively (Table 1).

The inpatient culture rate was significantly lower during the pandemic period compared with the pre-pandemic period in the Indian hospital (20.2/1000 patient-days during the pandemic period vs. 35.1/1000 patient-days during the pre-pandemic period; *p* < 0.001). In the USA, Hospital B had a significantly lower inpatient culture rate during the pandemic period compared with the pre-pandemic period (147.7/1000 patient-days vs. 180.2/1000 patient-days; *p* < 0.001), whereas in Hospital A it was significantly higher (276.8/1000 patient-days vs. 196.8/1000 patient-days; *p* < 0.001).

When examined according to specimen type, the respiratory culture rate was significantly higher in all three hospitals during the pandemic period compared with the pre-pandemic period (Indian hospital: 4.7/1000 patient-days vs. 4.3/1000 patients-days; *p* < 0.001; Hospital A: 31/1000 patient-days vs. 11.4/1000 patient-days; *p* < 0.001; Hospital B: 21.6/1000 patient-days vs. 15.8/1000 patient-days; *p* = 0.006), whereas the urine culture rate was significantly lower in all three hospitals (Indian hospital: 5.5/1000 patient-days vs. 14.6/1000 patient-days; *p* < 0.001; Hospital A: 19.9/1000 patient-days vs. 28.5/1000 patient-days; *p* < 0.001; Hospital B: 24.0/1000 patient-days vs. 30.0/1000 patient-days; *p* = 0.035) during the pandemic period compared with the pre-pandemic period. The blood culture rate was significantly lower during the pandemic period compared with the pre-pandemic period in the Indian hospital (10.1/1000 vs. 16.2/1000 patient-days; *p* < 0.001) and Hospital B (102.1/1000 vs. 134.3/1000 patient-days; *p* < 0.001), whereas it was significantly higher in Hospital A (224.8/1000 vs. 157/1000 patients-days; *p* < 0.001).

### 2.1. Culture Positivity Proportion

The blood culture positivity proportion after excluding contaminants was significantly higher during the pandemic period compared with the pre-pandemic period in the Indian hospital (19.6% (88/448) vs. 7.4% (233/3133); *p* < 0.001) and US Hospital A (15.1% (198/1311) vs. 9.0% (2772/30,775); *p* < 0.001), whereas no significant change was observed in Hospital B (5.8% (22/379) vs. 7.2% (2319/32,273); *p* = 0.38) (Table 2).

The respiratory culture positivity proportion was significantly higher during the pandemic period in the Indian hospital (39.6% (82/207) vs. 16.8% (138/823); *p* < 0.001), whereas no significant change was observed in either of the two US hospitals (Hospital A: 36.5% (66/181) vs. 29.5% (656/2224); *p* = 0.10; Hospital B: 18.6% (15/80) vs. 18.1% (687/3798); *p* = 0.89). The proportion of positive urine cultures was significantly higher during the pandemic period in the Indian hospital (29.3% (71/242) vs. 18.8% (528/2807); *p* < 0.001), whereas no significant change was observed in either of the two US hospitals (Hospital A: 49.1% (57/116) vs. 44.2% (2466/5576); *p* = 0.43; Hospital B: 48.3% (43/89) vs. 53.4% (3825/7212); *p* = 0.54) (Table 2).

### 2.2. Isolated Organisms

The proportion of coagulase-negative *Staphylococci* (CONS) as a contaminant in the blood cultures was significantly higher during the pandemic period in all three hospitals (Indian hospital: 1.8% (8/448) vs. 0.5% (15/3133); *p* = 0.003; Hospital A: 3.2% (42/1311) vs. 1.6% (493/30,775); *p* = 0.002; Hospital B: 4% (15/379) vs. 1.3% (405/32,273); *p* < 0.001) (Table 2). During the pandemic period, CONS was the organism most frequently isolated in blood cultures (after excluding CONS in single blood cultures) among all three hospitals (Table 3). 

During the pre-pandemic period, *Escherichia coli* was the organism most frequently isolated in blood cultures in the Indian hospital and in Hospital B, whereas *Staphylococcus aureus* was the organism most frequently isolated in blood cultures in Hospital A. *Klebsiella pneumoniae* was the organism most frequently isolated in respiratory cultures during the pandemic and pre-pandemic periods in the Indian hospital. In Hospital A, *Pseudomonas aeruginosa* was the most common organism isolated in respiratory cultures during the pandemic period, while *S. aureus* was the most common organism isolated during the pre-pandemic period. In Hospital B, *S. aureus* was the most common organism isolated in respiratory cultures during the pandemic period, while *P. aeruginosa* was the organism most frequently isolated during the pre-pandemic period. *E. coli* was the organism most frequently isolated in urine cultures during the pandemic and pre-pandemic periods in all three hospitals (Table 3).

### 2.3. Antimicrobial Resistance Proportion

The proportion of MDROs in blood cultures, after excluding common skin contaminants, was significantly higher during the pandemic period in the Indian hospital (6.9% (30/448) vs. 3.0% (93/3133); *p* < 0.001) and in Hospital A in the US (4.4% (57/1311) vs. 2.6% (804/30,775); *p* < 0.001), whereas no MDROs were isolated in blood cultures in Hospital B during the pandemic period (0.0% (0/379) vs. 1.2% (380/32,273) in the pre-pandemic period; *p* = 0.025) (Table 4).

The proportion of MDROs in respiratory cultures was significantly higher during the pandemic period in the Indian hospital (23.2% (48/207) vs. 9.0% (74/823); *p* < 0.001), but not in either of the two US hospitals (Hospital A: 12.7% (23/181) vs. 11.8% (262/2224); *p* = 0.73; Hospital B: 7.5% (6/80) vs. 5.0% (189/3798); *p* = 0.32). The MDRO proportion was significantly higher in urine cultures during the pandemic period in the Indian hospital (21.5% (52/242) vs. 14.0% (394/2807); *p* = 0.004) and in Hospital A (19.0% (22/116) vs. 12.3% (683/5576); *p* = 0.040), but no difference was observed in Hospital B (11.2% (10/89) vs. 14.8% (1067/7212); *p* = 0.39) (Table 4).

In the Indian hospital, no significant change in the antimicrobial resistance proportion was observed among the organisms most frequently isolated from blood cultures during the pandemic and pre-pandemic periods. However, in the Indian hospital, the proportion of carbapenem resistance was significantly higher during the pandemic period compared with the pre-pandemic period among *K. pneumoniae* isolates (34.4% (11/32) vs. 4.8% (2/41); *p* = 0.001) and *Pseudomonas aeruginosa* isolates (26.32% (5/19) vs. 0% (0/30); *p* = 0.003) in respiratory cultures (Table 3). In the Indian hospital, the proportion of carbapenem resistance was significantly higher during the pandemic period among *E. coli* (26.3% (10/38) vs. 8.6% (30/349); *p* = 0.002) in urine cultures (Table 4).

In the US, the proportion of methicillin-resistant *S. aureus* (MRSA) in blood cultures was significantly higher during the pandemic period in Hospital A (67.7% (44/65) vs. 47.3% (455/963); *p* = 0.020), but otherwise no significant changes were observed in the antimicrobial resistance proportion among other organisms during the pandemic compared with the pre-pandemic period (Table 4). Similarly, no significant changes were observed in the antimicrobial resistance proportion among organisms in urine and respiratory cultures during the pandemic compared with the pre-pandemic period in either of the two US hospitals.

## 3. Discussion

Our study results indicate that there are both similarities and differences in the microbiological results from the Indian and US hospitals before and during the COVID-19 pandemic. The respiratory culture rate was significantly higher during the pandemic period in all three hospitals. This is not surprising, as COVID-19 is primarily a respiratory illness; respiratory cultures were probably obtained on admission or during their hospital stay to rule out co-infections or secondary infections. In contrast to the blood culture rate, the urine culture rate decreased during the pandemic period in all three hospitals. However, the blood culture rate was significantly lower in the Indian hospital and Hospital B, whereas it was significantly higher in Hospital A during the pandemic period compared with the pre-pandemic period. The proportion of CONS as a contaminant in blood cultures significantly increased during the pandemic in all three hospitals. This observation is consistent with those of other laboratory-based studies conducted in several countries [12,13,14,15,16,20]. The increase in CONS as a contaminant in blood cultures could be attributed to the difficulty in performing venipunctures while using unfamiliar personal protective equipment (PPE).

Regarding the isolation of MDROs on the WHO priority pathogen list, the proportion of carbapenem resistant *K. pneumoniae* and *P. aeruginosa* in respiratory cultures and the proportion of carbapenem-resistant *E. coli* in urine cultures significantly increased during the pandemic period in the Indian hospital. This is consistent with the findings of other studies from India, which report high rates of carbapenem resistance among *K. pneumoniae*, *A. baumannii*, and *P. aeruginosa* in COVID-19 patients [4,10]. This could be attributed to an increased use of carbapenems [4,21] among COVID-19 patients in Indian hospitals, as well as lapses in infection control practices [4]. A multi-center study in India involving ten hospitals during peak COVID-19 activity reported that 47% of COVID-19 patients received carbapenems, and that carbapenems were the most frequently used antibiotics, accounting for approximately 20% of total antibiotic use during the study months [4]. Another study, which examined national antibiotic sales between 2018 and 2020, indicated a dramatic increase in the utilization of carbapenems during the peak months of COVID-19 activity [21]. Similar observations of increased carbapenem resistance among *K. pneumoniae* were observed during the pandemic period in Mexico [17] and Greece [18]. The proportion of MRSA in blood cultures in one of the US hospitals (Hospital A) was significantly higher during the pandemic period compared with the pre-pandemic period. Although we did not observe this in Hospital B, studies from the US involving pooled data from several hospitals have reported increased rates of healthcare-associated MRSA bloodstream infection during the COVID-19 period compared with the pre-pandemic period [22,23]. The increase in healthcare-associated MRSA bloodstream infections may be attributed to an increased utilization of central lines and mechanical ventilators among COVID-19 patients.

The variations in the prevalence of pathogens and antimicrobial resistance during the pre-pandemic and early pandemic periods in this study could be attributed to the enormous strain placed on the hospitals due to the COVID-19 pandemic. First, infection prevention and control measures were compromised due to shortages of PPE and qualified healthcare workers. For example, the lack of sufficient PPE resulted in the sharing or reusing of PPE, which might have led to cross contamination with MDROs [24]. Similarly, the shortage of healthcare workers led to long duty hours for existing staff, causing burnout and subsequently breaches in infection prevention and control measures, such as hand hygiene [25]. These challenges were exaggerated in LMICs, where the availability of infection prevention resources and qualified healthcare workers were limited even before the pandemic. Second, the pandemic led to the disruption of antimicrobial stewardship programs and the overuse of antibiotics for COVID-19 patients. The overuse of antibiotics for COVID-19 patients was more pronounced in LMICs than in HICs [26].

The strength of this study lies in the standardized methods that were used to analyze the data, which minimized the challenges associated with comparing data from different resource settings. However, there are some limitations. As this is a laboratory-based study, it is difficult to determine true infection versus colonization, especially from respiratory and urine cultures. Secondly, we did not examine the antibiotic utilization or infection control practices, which might have explained the significant increase in antimicrobial resistance proportion in the Indian hospital during the pandemic period. Finally, unlike the two US hospitals, the Indian hospital became a COVID-19 hospital, and this may have impacted its patient population demographics during the pandemic period.

In conclusion, when compared with the pre-COVID period, we observed a significant increase in the proportion of CONS as a contaminant in blood cultures among COVID-19 patients in both the Indian hospital and the two US hospitals. We also observed a significant increase in the proportion of Gram-negative MDROs from the WHO priority pathogen list in the Indian hospital and in the proportion of MRSA in one US hospital. These findings could be attributed to challenges with infection control practices and inappropriate use of antimicrobials during the first phase of the COVID-19 pandemic. Continuity of antimicrobial stewardship activities and better infection prevention measures are critical to optimize outcomes and minimize the burden of antimicrobial resistance among COVID-19 patients.

## 4. Methods

### 4.1. Study Description

This retrospective study was conducted by obtaining microbiology data from The Rural Development Trust (RDT) Hospital, Bathalapalli, Andhra Pradesh, a rural community hospital in India, and two community hospitals (Hospital A and Hospital B) in the BJC Healthcare System, St. Louis, MO, USA. The Indian hospital has 240 beds and provides services to both adults and children. The US hospitals provide services only to adults and have a bed capacities of 475 (Hospital A) and 426 (Hospital B). The study was restricted to patients ≥ 18 years old. This study was approved by Washington University School of Medicine (WUSM) in St. Louis, MO, USA and the Human Research Protection Office and RDT Hospital in India.

### 4.2. Data Collection

The microbiology data for the Indian hospital were obtained from 1 January 2017 to 31 December 2019 (pre-pandemic period) and from 15 April 2020 to 31 October 2020 (pandemic period), whereas for the two US hospitals, data were obtained from 1 January 2017 to 31 December 2019 (pre-pandemic period) and from 15 April 2020 to 30 September 2020 (pandemic period). For this study, we included all blood, respiratory, and urine cultures collected from inpatients who were admitted for more than 48 h. The Indian hospital was declared a COVID-19 hospital by the State Government of Andhra Pradesh and catered exclusively to COVID-19 patients from 15 April 2020 to 31 October 2020, whereas the two hospitals in the USA provided care to both COVID-19 and non-COVID-19 patients. All data from the two US hospitals were restricted to patients admitted with COVID-19 during the pandemic period (15 April 2020 to 31 August 2020) with a hospital stay of at least 48 h. For the Indian hospital, data were restricted to patients admitted with COVID-19 between 15 April 2020 and 25 October 2020 with a hospital stay of at least 48 h. We included two extra months (September 2020 and October 2020) of data for the Indian hospital to maximize the number of COVID-19 patients.

### 4.3. Organism Identification and Antimicrobial Susceptibility Testing

At the Indian hospital, microorganism identification was performed using matrix-assisted laser desorption ionization time-of-flight mass spectrometry (MALDI-TOF-MS) and antimicrobial susceptibility testing (AST) was performed using the Kirby–Bauer disk diffusion method as per Clinical Laboratory Standards Institute guidelines. The microbiology services for Hospital A are outsourced to a tertiary care hospital within the BJC Healthcare System which utilizes MALDI-TOF-MS for microorganism identification and the Kirby–Bauer disk diffusion method for AST. At Hospital B, VITEK-2 (bioMérieux, Marcy-l’Étoile, France) is used for microorganism identification and AST. Blood cultures are processed using automated blood culture systems at all three hospitals.

### 4.4. Statistical Analysis

Categorical variables were compared using Fisher’s exact or Chi square tests. Mann–Whitney U-tests were utilized for continuous variables, and rates were compared using Poisson regression. For all three hospitals, patient census data, including patient-days, were obtained from the hospitals’ administrative databases. A patient-day is a unit of measure denoting services rendered to one patient in an inpatient unit by midnight. Data de-duplication was performed by excluding identical organisms in the same type of culture (i.e., blood, respiratory, and urine) within an individual in a 14-day period. Organisms isolated in the same patient from two different culture types (e.g., blood vs. urine) were considered distinct. Blood culture contamination was defined as the isolation of one or more common commensal organism listed on the National Healthcare Safety Network of the Centers for Disease Control and Prevention 2022 list in only a single blood culture in one set, or in one of a series of two or more blood cultures [27]. For the respiratory cultures, isolation of normal oropharyngeal flora was not considered a positive culture. For the urine cultures, the growth of any organism was considered a positive culture. MDROs were defined according to European and US CDC standards, as previously described [28]. All statistical analyses were performed using SAS version 9.4 (Cary, NC, USA).

## Figures and Tables

**Table 1:** Inpatient cultures before and during the COVID-19 pandemic in the Indian hospital and the two US hospitals.

	Indian Hospital			Hospital A, USA			Hospital B, USA		
	COVID-19 Period (15 April 2020 to 31 October 2020)	Pre-COVID-19 Period (1 January 2017 to 31 December 2019)	*p*	COVID-19 Period (15 April 2020 to 30 September 2020) *	Pre-COVID-19 Period (1 January 2017 to 31 December 2019)	*p*	COVID-19 Period (15 April 2020 to 30 September 2020) *	Pre-COVID-19 Period (1 January 2017 to 31 December 2019)	*p*
Total number of inpatient cultures	897	6763		1608	38,555		548	43,283	
Patient-days **	44,380	192,935		5831	195,920		3711	240,258	
Cultures per 1000 patient-days	20.2	35.1	<0.001	275.6	196.8	<0.001	147.7	180.2	<0.001
Blood cultures (inpatient)	448	3133		1311	30,775		379	32,273	
Blood cultures per 1000 patient-days	10.1	16.2	<0.001	224.8	157	<0.001	102.1	134.3	<0.001
Urine cultures (inpatient)	242	2807		116	5576		89	7212	
Urine cultures per 1000 patient-days	5.5	14.6	<0.001	19.9	28.5	<0.001	24	30	0.035
Respiratory cultures (inpatient)	207	823		181	2224		80	3798	
Respiratory cultures per 1000 patient-days	4.7	4.3	<0.001	31.0	11.4	<0.001	21.6	15.8	0.006

* Data from the two US hospitals were restricted to admissions between 15 April 2020 and 31 August 2020. However, cultures for those patients who were admitted before 31 August were included if obtained by 30 September 2020. ** A patient-day is the unit of measure denoting services rendered to one patient in an inpatient unit by midnight.

**Table 2:** Proportion of positive cultures among blood, respiratory, and urine samples before and during the COVID-19 pandemic in the Indian hospital and the two US hospitals.

	Indian Hospital			Hospital A, USA			Hospital B, USA		
	COVID-19 Period (15 April 2020 to 31 October 2020) % Positive	Pre-COVID-19 Period (1 January 2017 to 31 December 2019) % Positive	*p*	COVID-19 Period (15 April 2020 to 30 September 2020) * % Positive	Pre-COVID-19 Period (1 January 2017 to 31 December 2019) % Positive	*p*	COVID-19 Period (15 April 2020 to 30 September 2020) * % Positive	Pre-COVID-19 Period (1 January 2017 to 31 December 2019) % Positive	*p*
Blood cultures after excluding contaminants	19.6% (88/448)	7.4% (233/3133)	<0.001	15.0% (197/1311)	9.0% (2772/30,775)	<0.001	5.8% (22/379)	7.2% (2319/32,273)	0.38
CONS contaminants in blood cultures	1.8% (8/448)	0.5% (15/3133)	0.003	3.2% (42/1311)	1.6% (493/30,775)	<0.001	4% (15/379)	1.3% (405/32,273)	<0.001
Other common skin contaminants in blood cultures	4.7% (21/448)	5.6% (176/3133)	0.43	0.5% (6/1311)	1% (297/30,775)	0.07	0.8% (3/379)	0.7% (239/32,273)	0.91
Respiratory cultures	39.6% (82/207)	16.8% (138/823)	<0.001	36.5% (66/181)	29.5% (656/2224)	0.10	18.6% (15/80)	18.1% (687/3798)	0.89
Urine cultures	29.3% (71/242)	18.8% (528/2807)	<0.001	49.1% (57/116)	44.2% (2466/5576)	0.43	48.3% (43/89)	53.0% (3825/7212)	0.54

* Data from the two US hospitals were restricted to admissions between 15 April 2020 and 31 August 2020. However, cultures for those patients who were admitted before 31 August were included if obtained by 30 September 2020.

**Table 3:** Top five organisms isolated from blood, respiratory, and urine cultures before and during the COVID-19 pandemic in the Indian hospital and the two US hospitals.

	Indian Hospital				Hospital A, USA				Hospital B, USA			
	COVID-19 Period (15 April 2020 to 31 October 2020) Organism % Positive		Pre-COVID-19 Period (1 January 2017 to 31 December 2019) Organism % Positive		COVID-19 Period (15 April 2020 to 30 September 2020) * Organism % Positive		Pre-COVID-19 Period (1 January 2017 to 31 December 2019) Organism % Positive		COVID-19 Period (15 April 2020 to 30 September 2020) * Organism % Positive		Pre-COVID-19 Period (1 January 2017 to 31 December 2019) Organism % Positive	
Top five organisms in blood cultures **	CONS *S. aureus* *K. pneumoniae* *E. coli* *E. faecium*	45.5% (40/88) 17.1% (15/88) 10.2% (9/88) 8.0% (7/88) 5.7% (5/88)	*E. coli* CONS *K. pneumoniae* *S. Typhi* *S. aureus*	27.5% (64/233) 15.5% (36/233) 10.3% (24/233) 6.9% (16/233) 5.2% (12/233)	CONS *S. aureus* *E. coli* *P. aeruginosa* *E. faecalis*	45.2% (89/197) 33.0% (65/197) 6.6% (13/197) 5.6% (11/197) 4.1% (8/197)	*S. aureus* CONS *E. coli* *E. faecalis* *K. pneumoniae*	35.5% (983/2772) 17.6% (487/2772) 13.1% (364/2772) 4.7% (129/2772) 4.4% (123/2772)	CONS *S. aureus* *E. faecalis* *K. pneumoniae* *Lactobacillus* sp.	59.1% (13/22) 13.6% (3/22) 9.1% (2/22) 9.1% (2/22) 4.6% (1/22)	*E. coli* *S. aureus* CONS *E. faecalis* *K. pneumoniae*	24.8% (575/2319) 18.2% (423/2319) 13.0% (302/2319) 11.3% (263/2319) 7.3% (169/2319)
Top five organisms in respiratory cultures **	*K. pneumoniae* *P. aeruginosa* *A. baumanni* *E. coli* *S. pneumoniae*	39.0% (32/82) 23.2% (19/82) 18.3% (15/82) 9.8% (8/82) 4.9% (4/82)	*K. pneumoniae* *P. aeruginosa* *E. coli* *S. aureus* *A. baumannii*	30.4% (21/138) 21.7% (30/138) 13.8% (19/138) 8.7% (12/138) 5.8% (8/138)	*P. aeruginosa* *S. aureus* *K. pneumoniae* *E. cloacae* *P. mirabilis*	39.4% (26/66) 28.8% (19/66) 7.6% (5/66) 4.6% (3/66) 4.6% (3/66)	*S. aureus* *P. aeruginosa* *K. pneumoniae* *S. maltophilia* *E. coli*	34.0% (223/656) 20.4% (134/656) 6.4% (42/656) 6.1% (40/656) 5.6% (37/656)	*S. aureus* *P. aeruginosa* *E. coli* *S. pyogenes*	60.0% (9/15) 33.3% (5/15) 6.7% (1/15) 6.7% (1/15)	*P. aeruginosa* *S. aureus* *S. maltophilia* *H. influenzae* *S. pneumoniae*	30.4% (209/687) 27.2% (187/687) 6.8% (47/687) 5.5% (38/687) 4.5% (31/687)
Top five organisms in urine cultures **	*E. coli* *K. pneumoniae* *Enterococcus* sp. *E. faecalis* *E. faecium*	53.5% (38/71) 21.1% (15/71) 18.3% (13/71) 4.2% (3/71) 2.8% (2/71)	*E. coli* *K. pneumoniae* *Enterococcus* sp. *E. faecalis* *Enterobacter* sp.	66.1% (349/528) 19.3% (102/528) 11.7% (62/528) 2.7% (14/128) 1.9% (10/528)	*E. coli* *P. mirabilis* *K. peumoniae* *Aerococcus urinae* *E. faecalis*	47.4% (27/57) 15.8% (9/57) 10.5% (6/57) 7.0% (4/57) 7.0% (4/57)	*E. coli* *K. pneumoniae* *P. mirabilis* *E. faecalis* Group B *Streptococcus*	47.3% (1166/2466) 12.0% (295/2466) 8.8% (217/2466) 6.8% (168/2466) 6.6% (162/2466)	*E. coli* *P. aeruginosa* *E. faecalis* *K. oxytoca* *K. pneumoniae*	44.2% (19/43) 21.0% (9/43) 9.3% (4/43) 7.0% (3/43) 7.0% (3/43)	*E. coli* *K. pneumoniae* *E. faecalis* *P. aeruginosa* *P. mirabilis*	47.0% (1798/3825) 12.3% (471/3825) 8.7% (334/3825) 7.6% (292/3825) 6.8% (259/3825)

* Data from the two US hospitals were restricted to admissions between 15 April 2020 and 31 August 2020. However, cultures for those patients who were admitted before 31 August were included if obtained by 30 September 2020. ** Can have >1 positive organism per culture and the denominator used is positive cultures.

**Table 4:** Overall MDRO proportion and proportion of MDROs on the WHO priority pathogen list among the three culture types during and pre-COVID-19 pandemic in the Indian and two US hospitals.

	Indian Hospital			Hospital A, USA			Hospital B, USA		
	COVID-19 Period (15 April 2020 to 31 October 2020) % Positive	Pre-COVID-19 Period (1 January 2017 to 31 December 2019) % Positive	*p*	COVID-19 Period (15 April 2020 to 30 September 2020) ** % Positive	Pre-COVID-19 Period (1 January 2017 to 31 December 2019) % Positive	*p*	COVID-19 Period (15 April 2020 to 30 September 2020) ** % Positive	Pre-COVID-19 Period (1 January 2017 to 31 December 2019) % Positive	*p*
% MDRO in blood cultures, excluding contaminants	6.7% (30/448)	3.0% (93/3133)	<0.001	4.4% (57/1311)	2.6% (804/30775)	<0.001	0.0% (0/379)	1.2% (380/32273)	0.025 *
% MDRO in respiratory cultures	23.2% (48/207)	9.0% (74/823)	<0.001	12.7% (23/181)	11.8% (262/2224)	0.73	7.5% (6/80)	5.0% (189/3798)	0.32
% MDRO in urine cultures	21.5% (52/242)	14.0% (394/2807)	0.004	19.0% (22/116)	12.3% (683/5576)	0.04	11.2% (10/89)	14.8% (1067/7212)	0.39
**Blood cultures** *E. coli* % 3GCR % CR *K. pneumoniae* % 3GCR % CR % MRSA % VRE % MR CONS	85.7% (6/7) 0.0% (0/7) 77.9% (7/9) 44.4% (4/9) 66.7% (10/15) 0.0% (0/11) 53.9% (28/52)	78.1% (50/64) 12.5% (8/64) 66.7% (16/24) 25.0% (6/24) 58.3% (7/12) 5.3% (1/19) 39.0% (21/54)	0.83 1.0 * 0.73 0.40 * 0.79 1.0 * 0.26	15.4% (2/13) 0.0% (0/13) 0.0% (0/5) 0.0% (0/5) 67.7% (44/65) 27.3% (3/11) 50.7% (45/89)	15.4% (56/364) 0.0% (0/364) 11.4% (14/123) 0.8% (1/123) 47.3% (455/963) 31.0% (62/200) 50.3% (245/487)	1.0 * 1.0 * 1.0 * 1.0 * 0.020 1.0 * 0.98	0.0% (0/0) 0.0% (0/0) 0.0% (0/2) 0.0% (0/2) 0.0% (0/3) 0.0% (0/2) 30.8% (4/13)	6.6% (38/575) 0.0% (0/575) 2.4% (4/169) 0.0% (0/169) 46.6% (197/423) 11.5% (34/296) 27.8% (84/302)	NE NE 1.0 * 1.0 * 0.25 * 1.0 * 0.82
**Respiratory cultures** *E. coli* % 3GCR % CR *K. pneumoniae* % 3GCR % CR % CR A. baumannii % CR P. aeruginosa % MRSA	87.5% (7/8) 37.5% (3/8) 65.6% (21/32) 34.4% (11/32) 73.3% (11/15) 26.3% (5/19) 66.7% (2/3)	100.0% (19/19) 26.3% (5/19) 64.3% (27/42) 4.8% (2/42) 62.5 (5/8) 0.0% (0/30) 41.7% (5/12)	0.76 0.66 * 0.94 0.001 0.77 0.003 0.57 *	50.0% (1/2) 0.0% (0/2) 20.0% (1/5) 0.0% (0/5) 0.0% (0/0) 23.1% (6/26) 36.9% (7/19)	24.3% (9/37) 0.0% (0/37) 19.1% (8/42) 0.0% (0/42) 0.0% (0/0) 26.9% (36/134) 55.2% (123/333)	0.45 1.0 * 1.0 * 1.0 * NE 0.73 0.30	0.0% (0/1) 0.0% (0/1) 0.0% (0/0) 0.0% (0/0) 0.0% (0/0) 20.0% (1/5) 44.4% (4/9)	22.2% (4/18) 0.0% (0/18) 5.3% (1/19) 0.0% (0/19) 100.0% (4/4) 16.3% (34/209) 70.6% (132/187)	1.0 * 1.0 * NE NE NE 1.0 * 0.14 *
**Urine cultures** *E. coli* % 3GCR % CR *K. pneumoniae* % 3GCR % CR % VRE	89.5% (34/38) 26.3% (10/38) 60.0% (9/15) 33.3% (5/15) 5.6% (1/18)	79.7% (278/349) 8.6% (30/349) 48.0% (49/102) 11.8% (12/102) 1.2% (1/81)	0.52 0.002 0.54 0.027 0.33 *	33.3% (9/27) 0.0% (0/27) 16.7% (1/6) 0.0% (0/6) 33.3% (2/6)	19.0% (222/1166) 0.3% (4/1166) 14.2% (42/295) 0.0% (0/295) 33.5% (80/239)	0.10 1.0 * 1.0 * 1.0 * 1.0 *	10.5% (2/19) 0.0% (0/19) 33.3% (1/3) 0.0% (0/3) 25.0% (1/4)	13.0% (234/1798) 0.1% (1/1798) 6.8% (32/471) 0.4% (2/471) 24.0% (104/434)	1.0 * 1.0 * 0.20 * 1.0 * 1.0 *

* A Fisher exact test was used to calculate *p*-values. 3GCR: third generation cephalosporin resistant; CR: carbapenem resistant; MDRO; multi-drug-resistant organism; MRSA: methicillin-resistant *S. aureus*; NE: not estimated; VRE: vancomycin resistant *Enterococci*; WHO: World Health Organization. ** Data from the two US hospitals were restricted to admissions between 15 April 2020 and 31 August 2020. However, cultures for those patients who were admitted before 31 August were included if obtained by 30 September 2020.

## Data Availability

All relevant data are contained within the article.
